# Using agrophotovoltaics to reduce carbon emissions and global rural poverty

**DOI:** 10.1016/j.xinn.2022.100311

**Published:** 2022-09-06

**Authors:** Huiming Zhang, Shan Liang, Kai Wu, Yueming (Lucy) Qiu, Yinyin Cai, Gabriel Chan, Shouyang Wang, Dequn Zhou, Yi Zhou, Zonghao Li

**Affiliations:** 1 School of Management Science and Engineering, Nanjing University of Information Science & Technology, Nanjing 210044, China; 2 School of Finance, Central University of Finance and Economics, Beijing 100081, China; 3 School of Public Policy, University of Maryland, College Park, MD 20742, USA; 4 Institute of Atmospheric Environmental Economics, Nanjing University of Information Science & Technology, Nanjing 210044, China; 5 Humphrey School of Public Affairs, University of Minnesota, Twin Cities, 301 S 19th Avenue S, Minneapolis, MN 55414, USA; 6 School of Economics and Management, University of Chinese Academy of Sciences, Beijing 100190, China; 7 Research Centre for Soft Energy Sciences, Nanjing University of Aeronautics and Astronautics, Nanjing 211100, China

## Introduction

Poverty-alleviation programs using solar energy (PAPSE) are poised to unlock unprecedented capital investments with significant potential to reconcile the energy–poverty–climate nexus.^1^ These programs are economically feasible because the costs of generating renewable energy have declined precipitously over the past decade; between 2010 and 2019, solar photovoltaic costs decreased by 82%. Furthermore, the number of annual equivalent-use hours of sunlight in most countries exceeds 1200, meaning that PAPSE approaches are feasible in many places and can be undertaken independently from the power grid.

However, programs that rely heavily on electricity sales without other collaborative means of making a profit result in only a limited increase in households’ incomes. Indeed, the degree to which such efforts succeed in reducing poverty has been largely over-estimated, with two key barriers holding things back. First, as photovoltaic power-generation technology has advanced, many countries—including Germany, Sweden, and the Netherlands—have started or begun planning to reduce or cancel solar photovoltaic feed-in-tariff subsidies. For example, China has imposed regulations on photovoltaic power programs to achieve grid parity, a critical consideration of the cost effectiveness for solar technologies.^2^

Second, land-use policies often limit the prospects for expanding photovoltaic power. Some countries restrict the land leasing or land acquisition needed to install photovoltaic panels at scale. Under Japan’s Agricultural Land Act, solar power is restricted from occupying agricultural land; a revised version of the regulation includes a requirement to dismantle facilities that have been found to have a negative impact on agricultural output. In India, land acquisition is one of the major challenges for large-scale projects above 100 MW. India’s approval process for land transactions is slow.

## APV programs

PAPSE projects are generally structured similarly to community solar programs and other jointly owned, renewable energy-generation projects developed in the United States and Europe. Traditional projects have sought to benefit low-income households by addressing energy poverty and/or by selling the electricity generated to the grid company. Such programs have been established in sub-Saharan Africa, including Nigeria, Kenya, Ghana, and Uganda.

**Figure 1:**
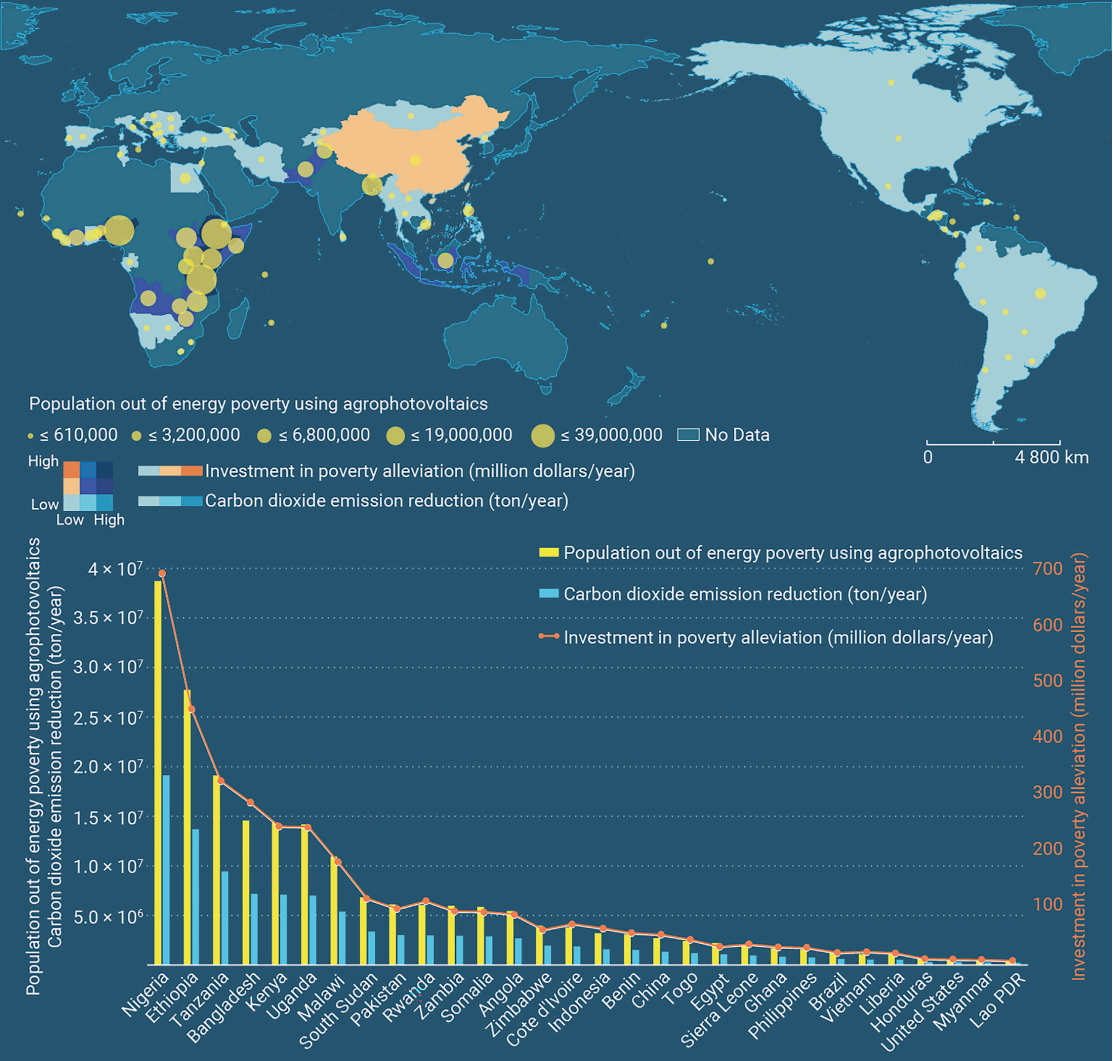
Performance and distribution of poverty alleviation programs using agrophotovoltaics The amount of investment, population out of energy poverty, and reductions in carbon dioxide emissions of poverty-alleviation programs in rural areas are estimated, excluding the agriculture sector’s effects on the co-benefits. The top 30 countries are listed.

As a new type of PAPSE project, agrophotovoltaics (APV) has the potential to address the limitations of PAPSE ones. APV programs that install solar panels above fishponds or over agriculture, flowers, fruit, or Chinese herbal medicine offer several notable merits. (1) Achieving ecological and climate benefits by integrating new energy power generation and the cultivation of agricultural (or aquicultural) products. (2) Deploying advanced photovoltaic technology to maximize energy production and address energy poverty. For example, perovskite cell technology has a photoelectric conversion efficiency that is greater than 18%.^3^ Our estimation suggests that the distribution of APV programs will reduce carbon dioxide emissions by 105 million tons and bring 212 million rural populations out of energy poverty annually. This estimate excludes the co-benefits for agriculture that are likely to emerge when the investment is established globally (Figure 1). (3) The shade under photovoltaic panels balances evapotranspiration and irrigation water. Such a setup can potentially increase land productivity by up to 70% and reduce water loss. (4) APV projects can distribute the co-benefits of photovoltaic power generation and agriculture more widely by selling electricity, leasing land, and enhancing agricultural-sector production of plants and/or fish. Research has demonstrated that implementing PAPSE policies in China increased rural per capita disposable income by 353 yuan per year.^4^ A typical project undertaken by the Zhongli Science and Technology Group, for example, has grown edible fungus under solar panels in Hebei Province. The project has led to an increase in annual per capita incomes to more than 7000 yuan.

APV programs are located in countries of all income levels. Projects can be found in the USA, Australia, and the UK and in developing countries including India, Lesotho, China, Vietnam, Malaysia, and Indonesia. However, only a few programs, such as those in Lesotho and China, are specifically used for poverty-alleviation purposes.^5^

## Four measures

We propose the following four measures to ensure the sustainable implementation of APV programs.

Use economic policy levers to fund APV-compatible agriculture/fishery. Diversified investment and financing strategies, such as public-private partnerships and build–operate–transfer methods, have been adopted to strengthen infrastructure in many poor areas. These investments in infrastructure are important because they help access agricultural markets. Similar approaches could be used to establish village development funds or cooperative finance to provide low-income households with multi-source financing services to purchase agricultural machinery and sell shade-sensitive products.

Relax land-use policies to improve rural land circulation. Rigorous land-lease regulations warrant a rethink. Land-lease licenses could be linked to farming products’ output or quality. This approach implies that a lease will be vacated if the yield, value, or quality level of products raised on a farm proves to be lower when solar panels are present than when panels are absent. For example, in Chiba-ken, Japan, an agriculture committee has such a provision, which is triggered if there is a 20% yield decrease in farm production. In line with this, governments should actively promote the circulation of collective, profit-oriented, rural construction lands and individual farmers' lands with confirmed land-operation rights. In South Korea, for example, subcontracting, joint-stock cooperation, and joint operation all contribute to rural land circulation.

Promote tailored, localized programs using information technologies. Programs that aim to alleviate poverty using APV should be developed according to geographical conditions, such as terrain, climate, and irrigation needs and capacity. Poor households, the government, and firms can use internet technologies to actively communicate with one another regarding the most promising crops for planting based on climate and soil quality. Recommendations might be to grow crops such as aloe in arid areas or mushrooms in humid, cool areas. We also suggest developing related e-commerce, expanding broadband “information superhighways” and wireless networks, and establishing “internet + logistics” systems to boost sales of APV farm produce. Another applicable technological innovation is a traceability system for APV farm produce and fish. Products can be affixed with a two-dimension code that contains information on the origin of a product and for quality assurance.

Develop flexible partnerships that link renewable energy firms, agriculture/fishery enterprises, and low-income communities. Renewable energy firms should be incentivized to establish photovoltaic power stations in rural areas. Poor households in these regions could benefit from related land rents and the wages they may earn from participating in farm and/or solar panel-related work with power companies’ ATV projects. Firms engaged in agriculture and aquaculture production, processing, e-commerce, tourism, and cultural activities should be integrated into wider efforts to promote such projects and to extend the stream of potential economic benefits. Renewable energy firms can establish strategic alliances that can advance these projects.

## References

[1] Casillas C.E., Kammen D.M. (2010). The energy-poverty-climate nexus. Science.

[2] Yan J.Y., Yang Y., Campana P.E. (2019). City-level analysis of subsidy-free solar photovoltaic electricity price, profits and grid parity in China. Nat. Energy.

[3] Wang F., Harindintwali J.D., Yuan Z.Z. (2021). Technologies and perspectives for achieving carbon neutrality. Innovation.

[4] Zhang H.M., Wu K., Qiu Y.M. (2020). Solar photovoltaic interventions have reduced rural poverty in China. Nat. Commun*.*.

[5] Ibrik I. (2020). Micro-grid solar photovoltaic systems for rural development and sustainable agriculture in Palestine. Agron.

